# Interferome signature dynamics during the anti-dengue immune response: a systems biology characterization

**DOI:** 10.3389/fimmu.2023.1243516

**Published:** 2023-08-10

**Authors:** Júlia Nakanishi Usuda, Desirée Rodrigues Plaça, Dennyson Leandro M. Fonseca, Alexandre H. C. Marques, Igor Salerno Filgueiras, Victor Gabriel Bastos Chaves, Anny Silva Adri, Amanda Torrentes-Carvalho, Mario Hiroyuki Hirata, Paula Paccielli Freire, Rusan Catar, Gustavo Cabral-Miranda, Lena F. Schimke, Guido Moll, Otavio Cabral-Marques

**Affiliations:** ^1^ Department of Clinical and Toxicological Analyses, School of Pharmaceutical Sciences, University of São Paulo, São Paulo, Brazil; ^2^ Interunit PostGraduate Program on Bioinformatics, Institute of Mathematics and Statistics, University of São Paulo, São Paulo, Brazil; ^3^ Departament of Immunology, Institute of Biomedical Sciences, University of São Paulo, São Paulo, Brazil; ^4^ Departament of Immunobiology, Institute of Biology, Federal Fluminense University, Niterói, Brazil; ^5^ Departament of Nephrology and Internal Intensive Care Medicine, Charité University Hospital, Berlin, Germany; ^6^ Department of Medicine, Division of Molecular Medicine, University of São Paulo School of Medicine, São Paulo, Brazil; ^7^ Laboratory of Medical Investigation 29, University of São Paulo School of Medicine, São Paulo, Brazil; ^8^ Network of Immunity in Infection, Malignancy, Autoimmunity (NIIMA), Universal Scientific Education and Research Network (USERN), São Paulo, SP, Brazil

**Keywords:** DENV, interferon, transcriptome, interferome, dengue

## Abstract

Dengue virus (DENV) infection manifests as a febrile illness with three distinct phases: early acute, late acute, and convalescent. Dengue can result in clinical manifestations with different degrees of severity, dengue fever, dengue hemorrhagic fever, and dengue shock syndrome. Interferons (IFNs) are antiviral cytokines central to the anti-DENV immune response. Notably, the distinct global signature of type I, II, and III interferon-regulated genes (the interferome) remains uncharacterized in dengue patients to date. Therefore, we performed an in-depth cross-study for the integrative analysis of transcriptome data related to DENV infection. Our systems biology analysis shows that the anti-dengue immune response is characterized by the modulation of numerous interferon-regulated genes (IRGs) enriching, for instance, cytokine-mediated signaling (e.g., type I and II IFNs) and chemotaxis, which is then followed by a transcriptional wave of genes associated with cell cycle, also regulated by the IFN cascade. The adjunct analysis of disease stratification potential, followed by a transcriptional meta-analysis of the interferome, indicated genes such as *IFI27*, *ISG15*, and *CYBRD1* as potential suitable biomarkers of disease severity. Thus, this study characterizes the landscape of the interferome signature in DENV infection, indicating that interferome dynamics are a crucial and central part of the anti-dengue immune response.

## Graphical Abstract

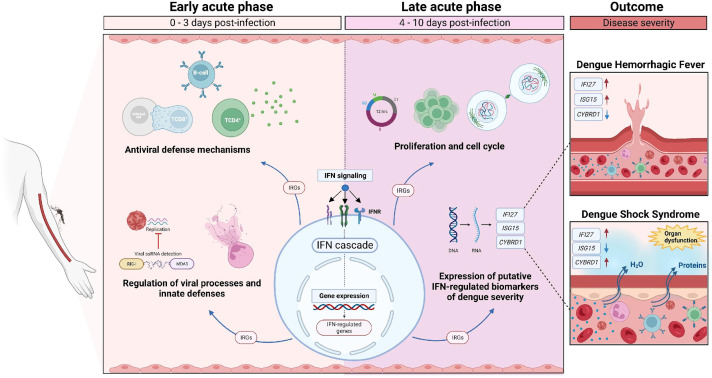

## 1 Introduction

Human arboviruses, such as the dengue, Zika, and chikungunya viruses, have frequently emerged or re-emerged worldwide in recent decades, and they are among the most epidemiologically essential viruses (1). Dengue virus (DENV) is the most prevalent among these (it affects a stunning 390 million individuals annually), and DENV is considered endemic in more than 100 countries (2). Surprisingly, despite its high prevalence and substantial global health impact, it is still characterized as a neglected tropical disease.

Four different DENV serotypes (DENV1-4) are transmitted by the vector *Aedes aegypti* and *Aedes albopictus* mosquitoes (3). The course of dengue infection can be divided into phases. The acute phase is characterized by high fever with abrupt onset, lasting between 2 to 7 days, which is characterized by multiple symptoms, such as malaise, myalgia, arthralgia, rash, retro-orbital pain, and headache (3, 4).

The acute phase can be classified into two stages: 1) Early acute (day 0 to 3 from symptom onset) and 2) Late acute or defervescence (day 4 to end of acute phase) (5). The critical stage during defervescence is characterized by increased capillary permeability and plasma leakage. This event may lead to severe shock, organ impairment, and bleeding. If the patient survives, the convalescent phase follows, where leaked fluids are reabsorbed and homeostasis is reestablished (4).

Moreover, dengue can be clinically classified by disease severity: 1) dengue fever (DF), characterized by fever and two or more dengue symptoms; 2) dengue hemorrhagic fever (DHF), marked by increased vascular permeability, plasma leakage, bleeding, and thrombocytopenia (3, 4); and 3) dengue shock syndrome (DSS), when DHF symptoms are aggravated with severe plasma leakage and circulatory compromise (6).

The DENV infection typically presents as a self-limiting febrile disease, indicating a central role of the immune system in controlling disease (7). Antigen-presenting cells can recognize viruses via pattern recognition receptors (PRRs), triggering the production of pro-inflammatory cytokines and phagocytic/microbicidal activity (8). This represents an initial step in controlling the virus spreading, followed by activation of the adaptive immune response (8, 9).

One key mechanism of the anti-viral immune response is the production of interferons (IFNs) by various cell types. IFNs are rapidly induced cytokines that respond strongly to DENV (10, 11). There are three families of IFNs: type I (mainly represented by IFN-α and IFN-β), type II (IFN-γ), and type III (IFN-λ1-4). IFNs are central mediators of innate and adaptive immunity as well as immune homeostasis.

For instance, interferon-alpha/beta (IFN-α/β)-binding to their receptor (IFNAR) triggers the activation of multiple downstream signaling pathways, including the canonical STAT1–STAT2–IFN-regulatory factor 9 (IRF9) signaling complex (12), which then binds to the IFN-stimulated response elements (ISREs) in gene promoters, leading to the induction of a large number of IFN-stimulated genes (ISGs).

Individual transcriptome studies of DENV-infected patients revealed that different IFN-associated genes are elicited in response to DENV infection (5, 6, 13–15) and that several of them are essential to promote a protective anti-viral reaction (5, 6), corroborated by linear and mechanistic approaches (16–18). In turn, DENV proteins antagonize IFN signaling (19, 20), underscoring the importance of these molecules in the response to DENV.

**Figure 1:**
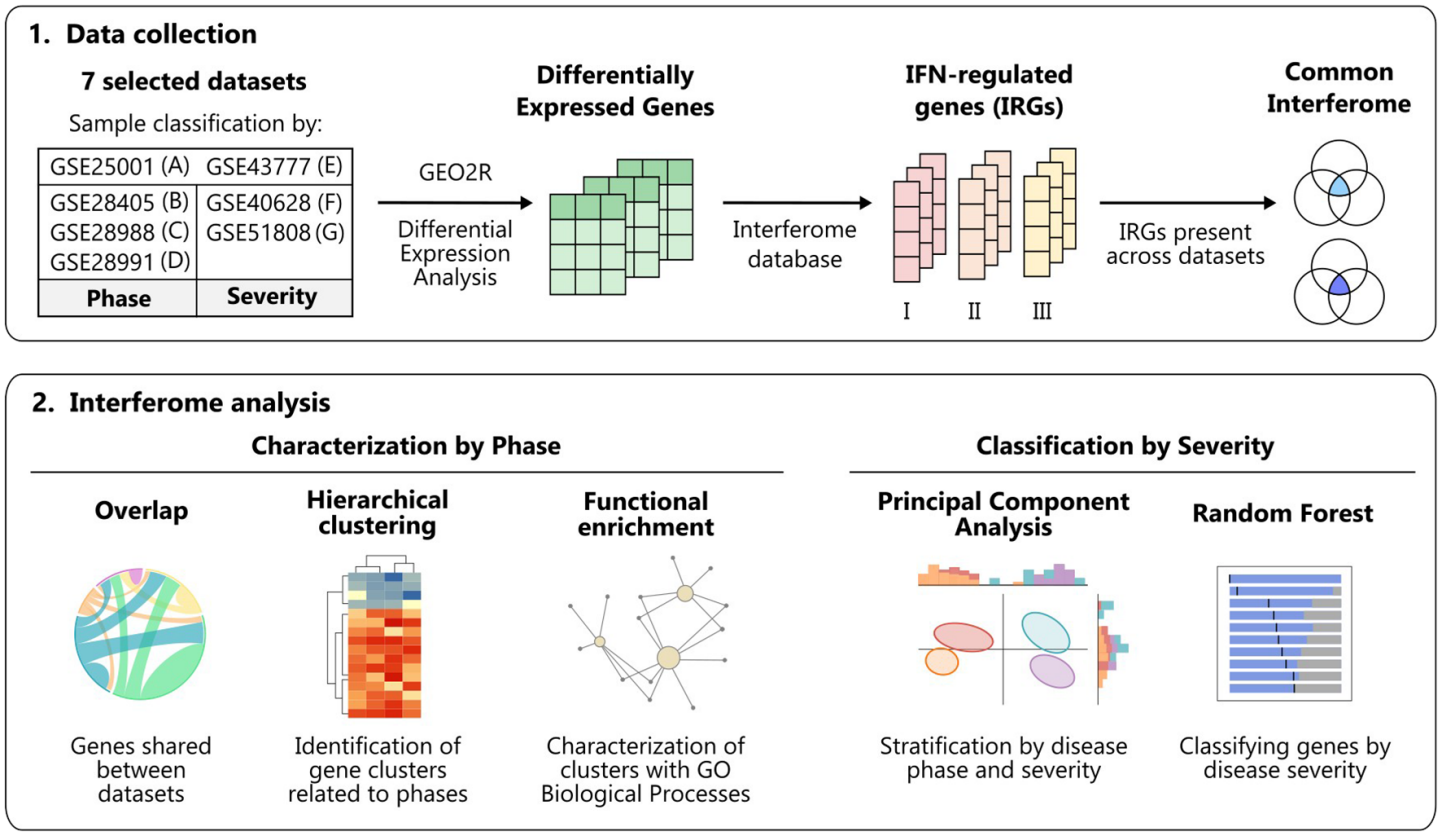
Workflow summary. Schematic overview of the data collection and analyses performed to characterize the interferome in dengue infection across disease phases and severity degrees. Figure created with Inkscape. *IFN, interferon; GO, Gene Ontology.*

A comprehensive analysis of the IFN signature in the context of dengue infection has not been conducted yet. Here, we performed an integrative analysis of multiple studies of dengue patients’ transcriptomes, considering the disease phases and severities, to characterize the dengue interferome [i.e., types I, II, and III interferon-regulated genes (IRGs)] (**Figure 1**).

## 2 Materials and methods

### 2.1 Data curation

**Figure 2:**
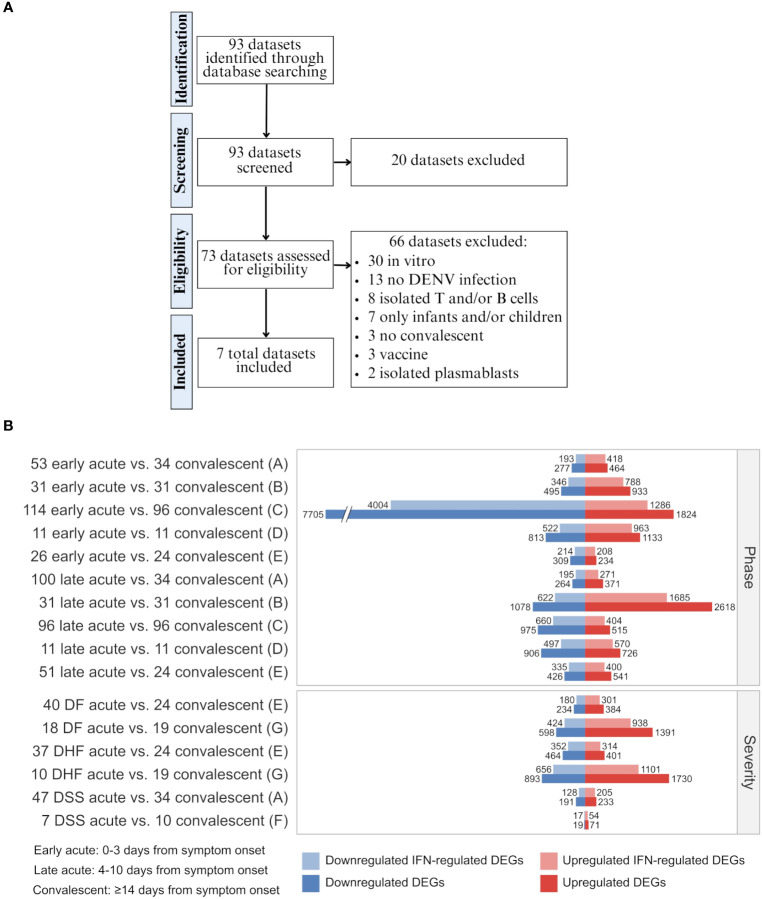
Data curation flow chart and differential expression of total genes and interferon-regulated genes across disease phases and disease severity degrees of dengue infection. **(A)** Steps of systematic search and assessment of datasets. Details of datasets and applied inclusion/exclusion criteria are available in **Table S1**. **(B)** Barplot showing the number of up- and downregulated DEGs and IFN-regulated DEGs for each cohort comparison and dataset as denoted by letters (A, GSE25001; B, GSE28405; C, GSE28988; D, GSE28991; E, GSE43777; F, GSE40628; F, GSE51808) (**Table S3**). The sample size of each cohort is indicated by a whole number in front of the group name. *DF, Dengue fever; DHF, Dengue hemorrhagic fever; DSS, Dengue shock syndrome; DEG, differentially expressed gene; IFN, interferon.*

We systematically searched the GEO database to collect publicly available gene expression data (https://www.ncbi.nlm.nih.gov/gds). Our search query *(“dengue virus”[MeSH Terms] OR “dengue virus”[All Fields] OR “DENV”[All Fields] OR “dengue”[MeSH Terms] OR “dengue”[All Fields]) AND “Homo sapiens”[Organism] AND (Expression profiling by high throughput sequencing[DataSet Type] OR Expression profiling by array[DataSet Type])* resulted in 93 series as of 17^th^ of August of 2021 (**Figure 2A**; All results in **Table S1**).

The inclusion criteria included: (1) *Homo sapiens* microarray or RNAseq expression data, (2) natural dengue infection, (3) blood or PBMC samples, (4) availability of convalescent-phase samples, and (5) at least one group with ten or more samples. The exclusion criteria included (1) *in vitro* samples, (2) cohorts with only infants and/or children, and (3) results from vaccine or drug trials. We included in our analysis seven datasets: GSE25001 (6, 21), GSE28405 (14, 22), GSE28988 (23), GSE28991 (24), GSE43777 (5, 25), GSE40628 (13, 26), and GSE51808 (15, 27).

The samples were categorized into three groups according to time after symptom onset: 1) Early acute (0-3 days), 2) Late acute (4-8 days), and 3) Convalescent (≥14 days). For dataset GSE43777, the late acute phase comprised days 4-10 from disease onset. When applicable, samples were also classified according to disease severity as: 1) Dengue fever (DF), 2) Dengue hemorrhagic fever (DHF), and 3) Dengue shock syndrome (DSS), as determined by the authors when samples were collected. Information about the included series is provided in **Table 1**.

**Table 1:** Study and sample information of included series.

Study	GEO accession	Sample size						Sample origin	Platform	Country	DENV serotypes	Ref.
		Early acute (0-3 days)	Late acute (4-8 days)*	Acute DF	Acute DHF	Acute DSS	Convalescent (≥ 14 days)					
A	GSE25001	53	100	–	–	47	34	Whole blood	Illumina HumanRef-8 v2 BeadChip	Vietnam	1, 2, unknown	(6, 21)
B	GSE28405	31	31	–	–	–	31	Whole blood	Sentrix HumanRef-8 Expression BeadChip	Singapore	1, 2, 3	(14, 22)
C	GSE28988	114	96	–	–	–	96	Whole blood	Illumina HumanRef-8 v3.0 expression BeadChip	Unknown	Unknown	(23)
D	GSE28991	11	11	–	–	–	11	Whole blood	Illumina HumanHT-12 V4.0 expression BeadChip	Unknown	Unknown	(24)
E	GSE43777	26	51	40	37	–	24	Whole blood	Affymetrix Human Genome U133 Plus 2.0 Array	Venezuela	1, 2, 3, 4	(5, 25)
F	GSE40628	–	–	–	–	7	10	Blood, PBMCs	SMD Print_980 LC-46	Vietnam	1, 2, unknown	(13, 26)
G	GSE51808	–	–	18	10	–	19	Whole blood	Affymetrix HT HG-U133+ PM Array Plate	Thailand	1, 2, 3, unknown	(15, 27)

Datasets, sample origin, and number of samples by disease phase and/or severity. Annotations: * 4-10 days in GSE43777. DF, Dengue fever; DHF, Dengue hemorrhagic fever; DSS, Dengue shock syndrome; DEG, differentially expressed gene; DENV, dengue virus; IFN, interferon.

### 2.2 Differential expression analysis

The identification of differentially expressed genes (DEGs) was performed with the GEO2R web tool (https://www.ncbi.nlm.nih.gov/geo/geo2r/) (28), applying statistical tests from the *limma* R package (29). All groups were compared to the convalescent. The DEGs were determined following the cutoffs of log2 fold-change (FC) > 1 (upregulated) or < -1 (downregulated) and adjusted p-value < 0.05, using R 4.0.5. DEGs and respective log2 FC are displayed in **Table S2**.

### 2.3 Interferome analysis

To specifically analyze the IFN network, we employed the Interferome database V2.01 (http://www.interferome.org/interferome/home.jspx) (30), which holds information about IFN-regulated genes (IRGs) expression after treatment with IFNs in various experimental systems. The DEGs were submitted to the database to identify IRGs and the specific regulating IFN type. The results summary and IRGs list are in **Tables S3**, **S4**.

### 2.4 Data integration and hierarchical clustering

The overlap of the IFN-regulated DEGs between datasets both by IFN type and disease phase were retrieved and represented with Circos (http://circos.ca/) (31) and UpSet plots generated with the web tool Intervene (https://asntech.shinyapps.io/intervene/) (32). The data used to create the plots are provided in **Tables S5**, **S6**. For each combination of IFN type and disease phase or severity, the genes shared by all the datasets considered were selected (**Table S7**). The heatmaps of the log2 fold-change of these shared genes across disease phases or severities were generated with the web tool Morpheus (https://software.broadinstitute.org/morpheus/) (33). The hierarchical clustering method utilized was Euclidean distance complete linkage. The data used to create the heatmaps are provided in **Tables S8**, **S9**.

### 2.5 Functional enrichment

Functional enrichment analysis based on the clusters of common genes was performed with the web tool EnrichR (https://maayanlab.cloud/Enrichr/) (34). The resulting enriched Gene Ontology (GO) Biological Processes (BPs) were filtered by adjusted p-value < 0.05, as well as combined scores and categories befitting the analyzed cell types. When many results from EnrichR were redundant or closely related terms, these were also filtered with Revigo (http://revigo.irb.hr/) (35). The most relevant BPs were represented with an alluvial plot generated with R package ggalluvial (36). The functional enrichment analysis according to disease phase and IFN type was conducted with R 4.0.5 and the ClusterProfiler (37) R package. The results were filtered by adjusted p-value < 0.05 and presented as dot plots and networks. The results from the functional enrichment analysis by cluster are available in **Table S10** and by disease phase and IFN type in **Table S11**.

### 2.6 Principal component analysis

Datasets A and E, which had the largest number of samples per phase and severity group (i.e., DSS and DHF, respectively), were selected to analyze the power of the interferome to discriminate the disease severities. The expression values were processed on the ExpressAnalyst web tool (https://www.expressanalyst.ca/) (38), and log2 normalized when applicable. For each disease phase, the IRGs previously identified as commonalities were selected. As of dataset E, four samples (GSM1071100, GSM1071104, GSM1071105, GSM1071108) identified as outliers in the PCA were removed from further analyses. Principal Component Analyses (PCA) by singular value decomposition (39, 40) were performed with the log2-transformed expression values of the intersection genes (**Table S12**) utilizing R 4.0.5 and packages factoextra (41), ggplot2 (42), and ggExtra (43).

### 2.7 Ranking of severity-classifying genes

The samples from datasets A and E were grouped by disease severity for each phase. The log2-transformed expression values of the commonalities used in the respective PCAs were applied in the random forest (RF), a machine learning algorithm, which uses the combination of multiple tree classifiers (44) to identify classifiers of dengue severity, using R 4.1.3 and package randomForest (45) (data used in RF available in **Table S13**). For cross-validation, 75% of the data was set for training and 25% for testing. A heatmap for each dataset depicting the fold change of the top 10 severity-classifying genes across disease phases was plotted using Morpheus (33) (data available in **Table S14**).

### 2.8 Transcriptional meta-analysis and gene annotation

The meta-significant genes were obtained by the Fisher p-value combination method using the ExpressAnalyst web tool (https://www.expressanalyst.ca/) (38) (results in **Table S15**). We used the late acute phase samples from datasets A and E to perform a meta-analysis. Briefly, the log2-transformed expression values were adjusted for batch effect with the combat function from R package sva (46) with R 4.1.3, and the differential expression analyses compared the severe dengue (DSS and DHF) samples with the non-severe dengue (non-DSS and DF) samples. We used Fisher’s method to obtain combined p-values for information integration, as recently described (47). A box plot was generated using the Robust Multi-array Average (RMA)-normalized and mean-summarized expression of the putative biomarkers for predicting severe dengue clinical outcome (*IFI27*, *ISG15*, and *CYBRD1)* genes from dataset G (GSE51808) (data available in **Table S16**). Wilcoxon’s test was applied to evaluate the statistical significance between acute DF or acute DHF and convalescent groups. The gene functions were retrieved from the NCBI Gene database (https://www.ncbi.nlm.nih.gov/gene/) (48) and UniProt Knowledgebase (https://www.uniprot.org/) (49) (**Table S17**).

## 3 Results

### 3.1 The interferome signature is a hallmark feature of the anti-dengue immune response

We performed a comprehensive multi-study analysis of dengue cohorts to characterize the interferome signature induced by DENV infection according to the disease phase. We obtained seven datasets of dengue fever transcriptomes according to our inclusion criteria of (1) *Homo sapiens* microarray or RNAseq expression data, (2) natural dengue infection, (3) blood or PBMC samples, (4) availability of convalescent-phase samples, and (5) at least one group with ten or more samples; and exclusion criteria of (1) *in vitro* samples, (2) cohorts with only infants and/or children, and (3) results from vaccine or drug trials. All these datasets were generated through microarray technology (GSE25001 (6, 21) (A), GSE28405 (14, 22) (B), GSE28988 (23) (C), GSE28991 (24) (D), GSE43777 (5, 25) (E), GSE40628 (13, 26) (F), and GSE51808 (15, 27) (G) (**Table 1**).

We compared the gene expression profiles of the different disease phases (early or late acute phases in contrast with the convalescent phase; datasets A-E) and severities (DF, DHF, or DSS acute phase in contrast with the convalescent phase; datasets A, E-G) through differential expression analyses (DEAs). Following, we identified the interferon-regulated genes (IRGs), as summarized in **Figure 2B** and **Table S3**. These results show that most DEGs induced by DENV are regulated by IFNs (On average 72% [minimum: 55%, maximum: 82%]), including a mix of downregulated and upregulated genes. These data confirm the well-established pivotal role of IFNs and the cascade of IFN-associated genes transcribed during the anti-dengue immune response.

### 3.2 Consistent IFN type I and II interferome signature during different dengue phases

**Figure 3:**
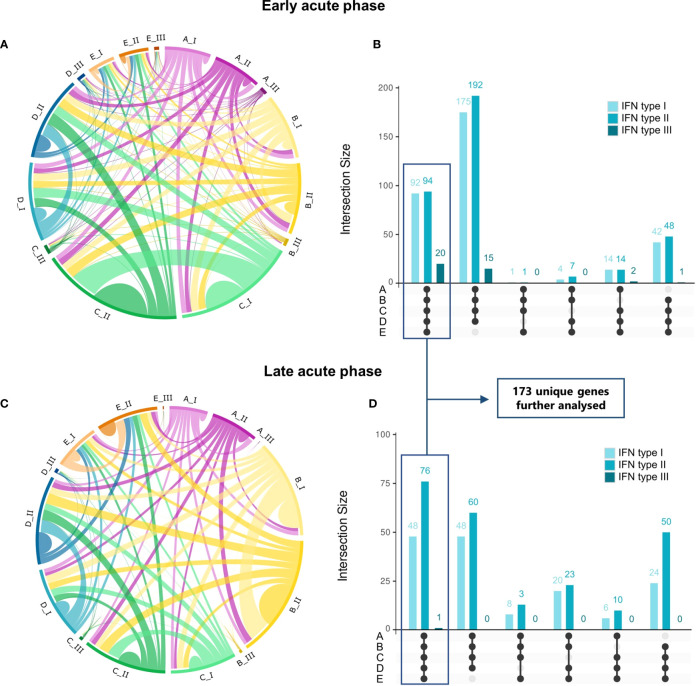
Commonalities and uniquenesses of interferon-regulated genes across studies by disease phase and IFN type. **(A, C)**, Circos plots representing IRGs regulated by IFN types I, II, or III that are shared across datasets in the early acute **(A)** or late acute **(C)** disease phases (**Table S5**). Colors represent the dataset [magenta, dataset A (GSE25001); yellow, dataset B (GSE28405); green, dataset C (GSE28988); blue, dataset D (GSE28991); orange, dataset E (GSE43777)], from lighter to darker indicating genes regulated by IFN types I, II or III, respectively. **(B, D)**, UpSet plots of the number of IRGs shared between datasets in the early acute **(B)** or late acute **(D)** disease phases. Bars are colored according to the regulating IFN type and indicate the number of genes shared in the dataset intersections denoted by connected black dots. Boxes highlight genes common to all datasets (commonalities), totalizing 173 unique genes after duplicate removal, which are further analyzed. A list of genes and complete intersections is available in **Tables S6**, **S7**, respectively. *IRG, interferon-regulated gene; IFN, interferon*.

We next analyzed the overlap between dengue’s early and late acute phases, identifying the transcriptional intersections between studies A to E. The investigation of common IRGs found 173 common DEGs between the datasets during early and late acute phases (**Figures 3A, C**, respectively, **Table S5**), thus revealing a substantial overlap between genes regulated by IFN type I and type II. In contrast, only a few IFN type III-regulated genes were identified. Intriguingly, the distinct difference in the number of IFN type III-regulated genes may be attributed to the limited experimental data available for IFN type III, which has been only recently characterized (50), resulting in fewer annotated genes on the database. In contrast, types I and II IFNs have been studied extensively (51).

We found a distinct interferome signature across the datasets during early and late acute phases (**Figures 3B, D**). This indicates that several IRGs are consistently modulated during the anti-DENV immune response. In the early acute phase, 92, 94, and 20 genes were found to be regulated by type I, II, and III IFNs, respectively, across the five datasets (commonalities). Likewise, in the late acute phase, 48, 76, and 1 gene(s) were found to be regulated by type I, II, and III IFNs, respectively. These commonalities in interferome signatures across datasets suggest a conserved response to DENV infection. A comprehensive list of these genes is provided in **Table S6**, and their intersection in **Table S7**.

### 3.3 Interferome clusterization at the early and late acute dengue phases

**Figure 4:**
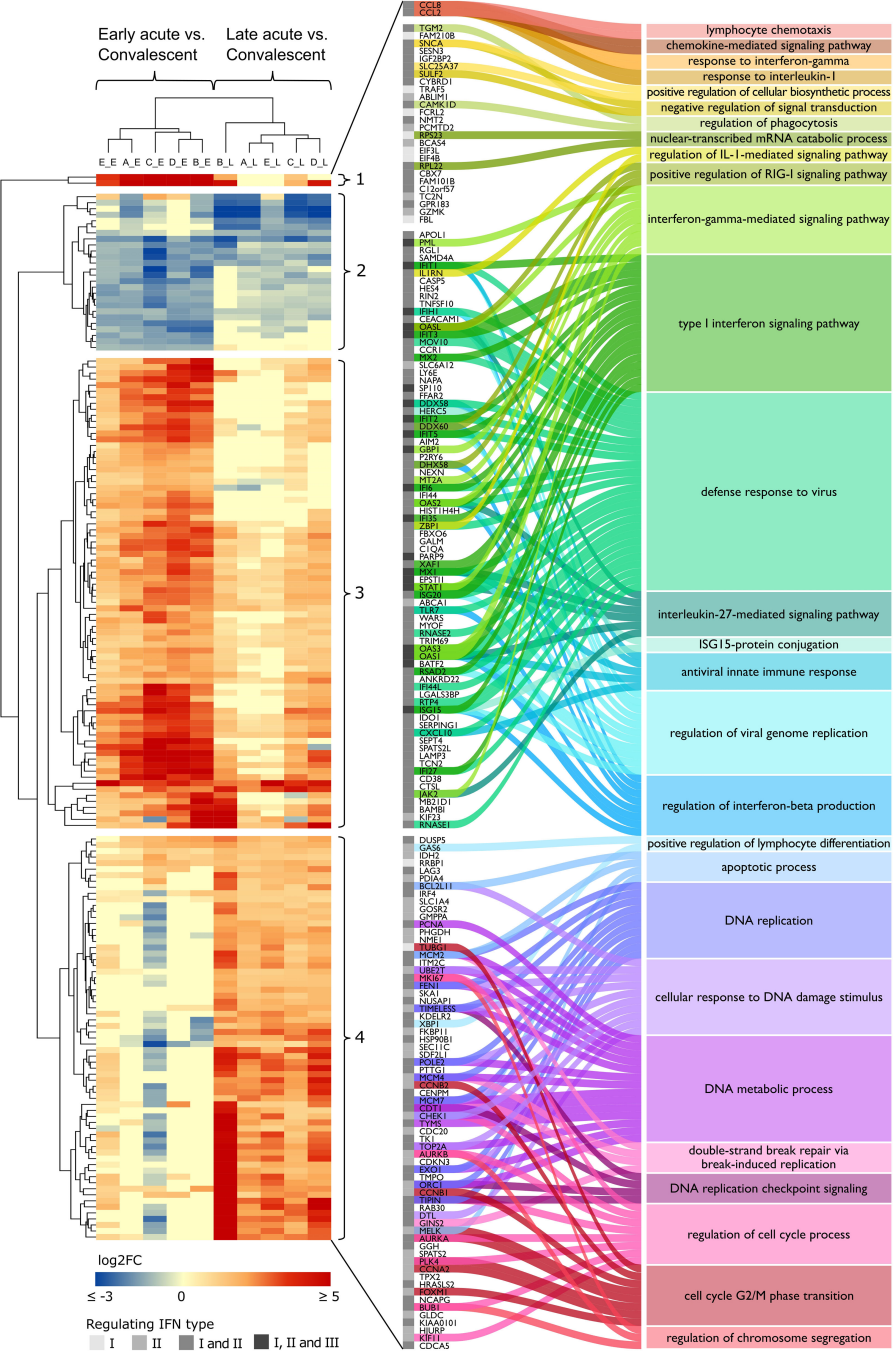
DENV infection acute phase interferome landscape. Heatmap of log2 FC of the common IRGs across the acute phases in datasets A to E (**Table S8**). The red color scale denotes up-regulated genes; the blue color scale denotes down-regulated genes, yellow color denotes genes not differentially expressed (FC close to zero or missing). Hierarchical clustering by Euclidean distance complete linkage metric discriminated cohorts (columns) by disease phase (early acute, late acute) and IRGs (rows) into four distinct clusters (1–4). The amplified view and alluvial plot represent the IRGs of each cluster and the main BPs associated with each cluster. The IFN types that regulate the IRGs are indicated by a grayscale column, from lighter to darker, representing IFN types I and II alone or I and II, as well as I, II, and III together. Complete functional enrichment results by cluster are available in **Table S10**. Heatmap columns legend: First letter indicates dataset (A, GSE25001; B, GSE28405; C, GSE28988; D, GSE28991; E, GSE43777), while second letter indicates disease phase comparisons (E, early acute vs. convalescent; L, late acute vs. convalescent). *DENV, dengue virus; FC, fold change; IRG, interferon-regulated gene; BP, biological process; IFN, interferon; IL-1, interleukin-1; RIG-I, retinoic acid-inducible gene I; ISG15, ISG15 ubiquitin-like modifier.*

We characterized the interferome expression patterns across the transcriptome datasets from dengue patients at acute phases (datasets A-E) with an unsupervised hierarchical clustering analysis (**Figure 4** and **Table S8**). The IRG expression pattern segregated patients at early acute from those at late acute phases. To further investigate whether the IRG expression pattern could also stratify the patients by disease severity, we analyzed samples from DF, DHF, and DSS patients. However, we found that the differences in the expression pattern of IRGs were not strong enough to segregate by disease severity (**Figure S1** and **Table S9**).

Next, we characterized the clusters through gene ontology (GO) analysis to identify distinct biological processes (BPs) that are enriched by the differential expression signatures found in our gene sets (**Figure 4** and **Table S10**). Namely, cluster 1, composed of strongly upregulated genes CCL8 and CCL2 during the early acute phase; cluster 2, comprising downregulated genes at early and late acute phases; cluster 3, including genes more upregulated in the early acute phase; and cluster 4, consisting of genes more upregulated in the late acute phase. Cluster 1 enriched BPs were related to the migration and chemotaxis of lymphocytes, granulocytes, and mononuclear cells. Cluster 2 included genes involved in phagocytosis, cellular response to catecholamine stimulus, and anion homeostasis. Cluster 3 exhibited genes enriching several processes related to the response to the virus (e.g., positive regulation of RIG-I signaling pathway, regulation of viral genome replication) and IFN-related processes (e.g., ISG15-protein conjugation, regulation of type I IFN production). Cluster 4 comprises genes enriching several cell cycle-associated processes such as phase transition, checkpoint, and DNA replication.

**Figure 5:**
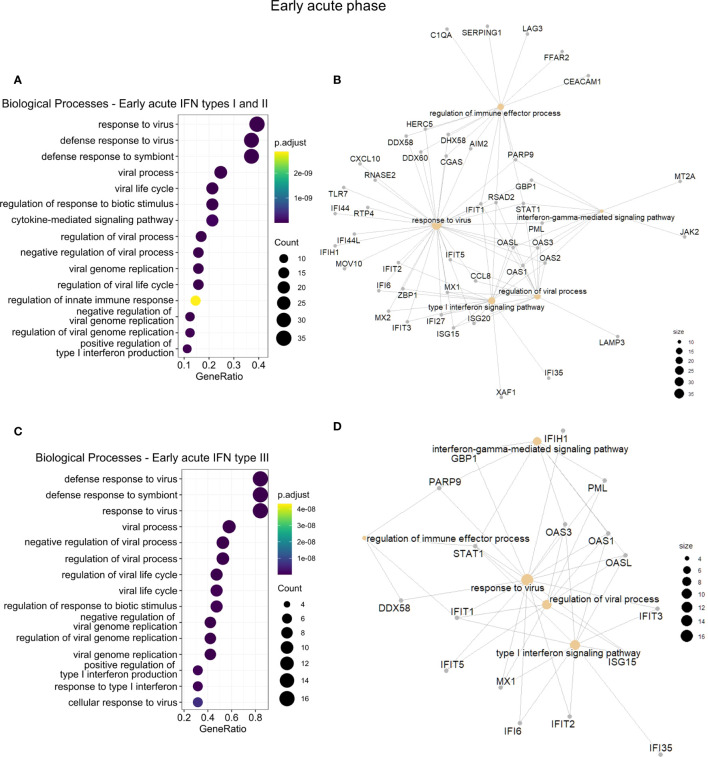
Functional gene enrichment characterization of the early acute phase interferome. **(A, C)**, Dot plots of the top 15 BPs enriched by the early acute IRGs regulated by IFN types I and II **(A)** or regulated by IFN type III **(C)**. The color scale indicates the adjusted p-value, and the dot size represents the number of input genes associated with the BP. **(B, D)**, Networks of the main enriched BPs (khaki nodes) and respective genes (gray nodes) for the early acute IRGs regulated by IFN types I and II **(B)** or regulated by IFN type III **(D)**. Node size represents the number of input genes associated with the BP. Complete functional enrichment results available in **Table S11**. *BP, biological process; IRG, interferon-regulated gene; IFN, interferon.*

**Figure 6:**
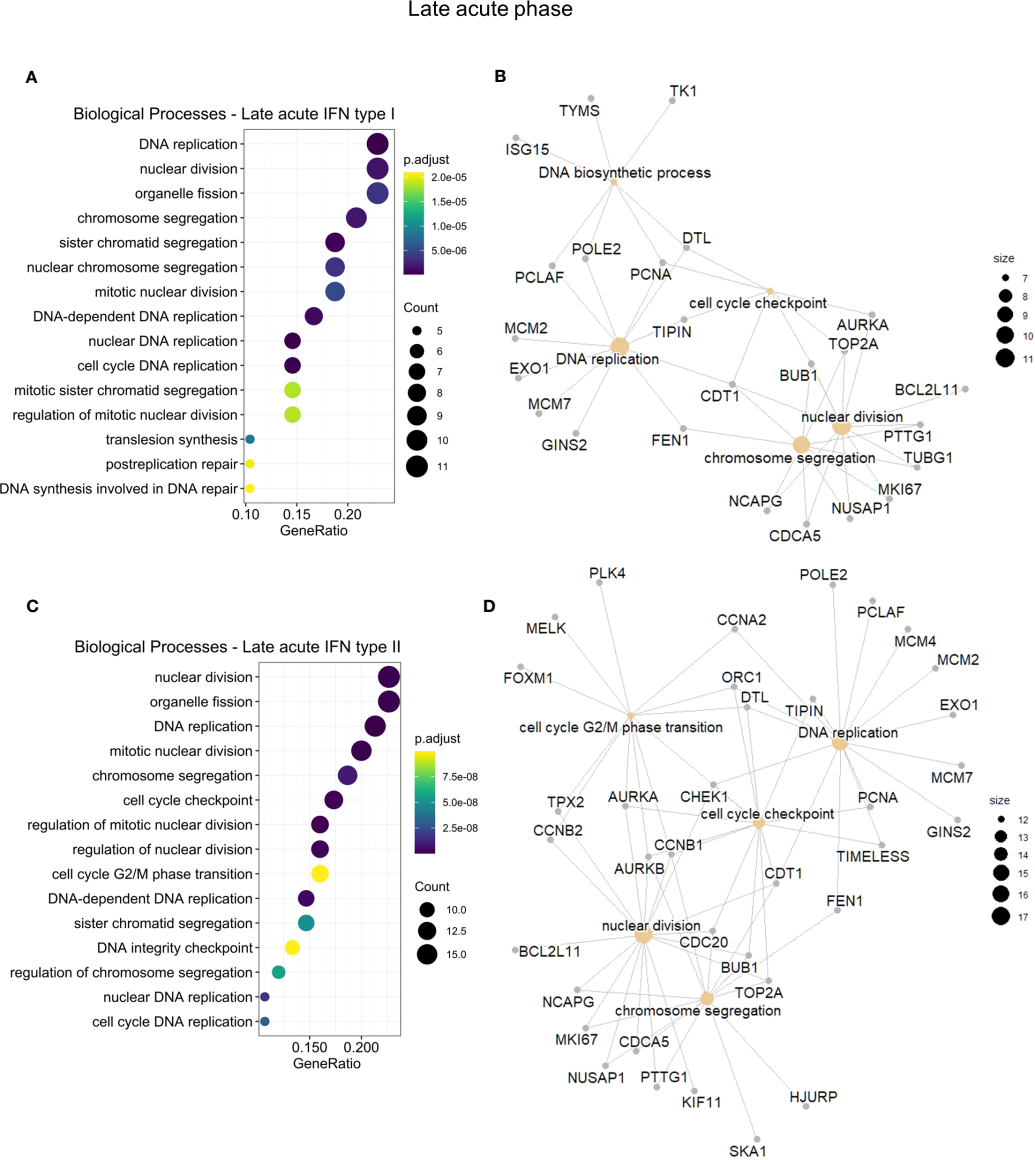
Functional gene enrichment characterization of the late acute phase interferome. **(A, C)**, dot plots of the top 15 BPs by gene ratio enriched by the late acute IRGs regulated by IFN types I **(A)** or regulated by IFN type II **(C)**. The dot color indicates the adjusted p-value, dot size represents the number of input genes associated with the BP. **(B, D)**, Networks of the main enriched BPs (khaki nodes) and respective genes (gray nodes) for the early acute IRGs regulated by IFN types I **(B)** or regulated by IFN type II **(D)**. Node size represents the number of input genes associated with the BP. Complete functional enrichment results available in **Table S11**. *BP, biological process; IRG, interferon-regulated gene; IFN, interferon.*

To assess whether GO differences depended on the particular IFN type, we further conducted a functional enrichment analysis of the 173 common IRGs by IFN type and disease phase (**Table S11**). In the early acute phase (**Figure 5**), genes regulated by IFN types I and II robustly overlapped (86 in 100 genes). Hence, we joined these genes and carried out the enrichment analysis. For the three IFN types, we found a predominance of BPs related to host regulation and defense against the virus, analogous to the enrichment results of cluster 3. In the late acute phase (**Figure 6**), considering that only one common gene was regulated by IFN type III, we performed the enrichment analysis only for types I and II. We found several cell cycle-associated BPs during the late acute phase, as seen in cluster 4 (**Figure 4**). Hence, these findings indicate a phase-specific interferome signature.

### 3.4 Anti-DENV interferome stratifies patients according to disease phase and severity

**Figure 7:**
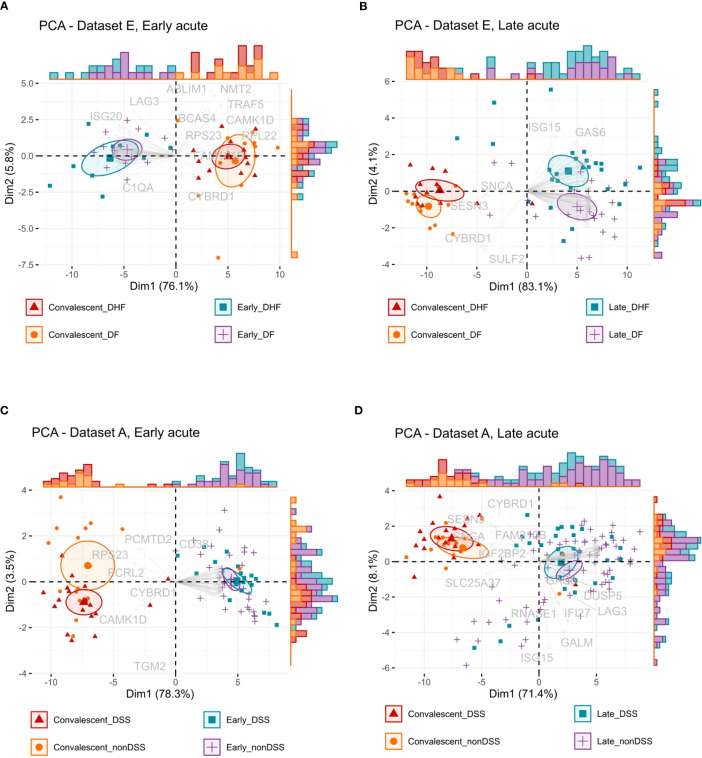
Interferon-regulated genes stratification capacity by disease phase and severity. **(A, B)**, Principal component analysis (PCA) biplots of the log2-transformed gene expression values from dataset E of the early acute phase **(A)** or late acute phase **(B)** IRGs common across all studies. **(C, D)**, PCA biplots of the log2-transformed gene expression values from dataset A of the early acute phase **(C)** or late acute phase **(D)** IRGs common across all studies. Data is available in **Table S12**. Ellipses represent the concentration of samples. Vectors represent the loadings, individual contributions of the genes. Histograms represent the distribution of samples across the biplot. Color and shape identify the groups: red color and triangles represent the convalescent more severe samples (DHF or DSS), orange color and dots represent the convalescent less severe samples (DF or nonDSS), blue color and squares represent the acute phase more severe samples (DHF or DSS), purple color and positive signs represent the acute phase less severe samples (DF or nonDSS). *IRG, interferon-regulated gene. DSS, Dengue shock syndrome DF; nonDSS, non-Dengue shock syndrome; Dengue fever; DHF: Dengue hemorrhagic fever.*

We further examined a possible stratification power of the anti-DENV interferome with principal component analysis (PCA), considering disease phases and severities. The PCA of IRGs indicated that they stratify DF, DHF, non-DSS, and DSS dengue patients at early and late acute phases from their counterparts in convalescence (**Figures 7A–D** and **Table S12**). In the late acute phase, the interferome signature could differentiate DF patients from DHF patients and partially distinguish non-DSS from DSS patients. However, for the IRGs identified in the early acute phase, there was only minimal differentiation power based on disease severity. This fact implies that the interferome signature at the later stage of DENV infection may play a distinct role compared to early dengue stages.

**Figure 8:**
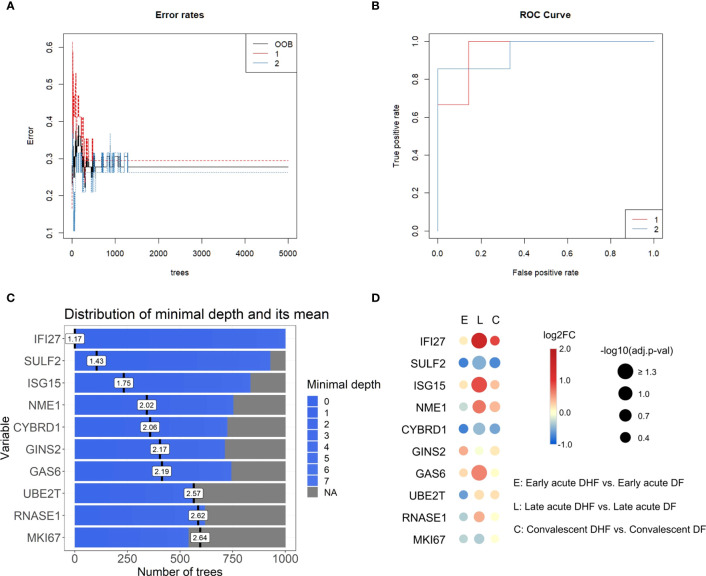
Top interferon-regulated genes for severity classification in the late acute phase ranked by random forest (Dataset E). **(A)**, Error rates of random forest models by number of trees. **(B)**, Receiver operating characteristic (ROC) curve of the generated classifying models. The red line corresponds to group 1 (late acute DF), blue line corresponds to group 2 (late acute DHF). **(C)**, Bar plot of the top ten severity classifying genes ranked by the random forest model, number of trees, and distribution of the minimal depth. Blue bars represent the minimum and maximum minimal depth, and black vertical lines represent the mean minimal depth for each classifying gene. Data input of log2-transformed expression values of IRGs common across datasets A to E in the late acute phase are available in **Table S13**. **(D)**, Bubble heatmap of the log2FC of IRGs resulting from the DHF vs. DF comparison for each disease phase (early acute, late acute, convalescent). The red scale indicates positive FC (up-regulated genes), blue scale indicates negative FC (down-regulated genes). Bubble size represents -log10-transformed adjusted p-value. Data is available in **Table S14**. *IRG, interferon-regulated gene; DHF, dengue hemorrhagic fever; DF, dengue fever; FC, fold chang*e, *OOB: out-of-bag.*

To better understand the interferome’s stratification power for dengue patients according to disease severity, we next applied random forest (RF), a machine learning algorithm, to identify classifiers of dengue severity (**Table S13**). In agreement with the PCA results, RF analysis of DF versus DHF late acute groups indicated an out-of-bag (OOB) error rate of 27.78% and area under the curve (AUC) of the receiver operating characteristic (ROC) curves of 0.952 (**Figures 8A, B**). Thus, IRGs are strong classifiers of DHF at the late phase. However, we did not find solid classifiers for DSS or the early acute phase. For dataset A, comparing non-DSS and DSS late acute groups, the OOB error rate was high for the non-DSS group (group 1), and the AUC of the ROC curves was 0.833 (**Figures S2**). When we performed RF with early acute samples from dataset E and dataset A, the OOB error rate was 31.58% and 33.33%, with AUC of 0.583 and 0.622, respectively (**Figure S3**).

The top ten IRGs classifying dengue severity (DF and DHF; **Figure 8C**) were *SULF2*, *ISG15*, *NME1*, *CYBRD1*, *GINS2*, *GAS6*, *UBE2T*, *RNASE1*, and *MKI67*, in decrescent order. We evaluated the expression patterns of these top 10 genes through DEAs within each disease phase, comparing disease severities (**Figure 8D** and **Table S14**). Only *IFI27* and *ISG15* were significantly differentially expressed between DHF and DF samples in the late acute phase. To further investigate whether these ten genes could also classify by disease severity, including DSS, we performed a meta-analysis with the late acute samples of datasets A and E. We found 872 meta-significant genes (**Table S15**). Of the top ten ranked genes, *IFI27*, *ISG15*, and *CYBRD1* were also identified among the meta-significant genes. To further validate these genes as putative biomarkers for predicting severe dengue clinical outcome classification across disease phases, we analyzed their expression on an independent cohort (dataset G). The acute and convalescent phases were significantly different, while there was no difference between the convalescent and healthy control samples (**Figure S4** and **Table S16**).

## 4 Discussion

The interferome is a highly complex ancient molecular system (already present in jawed vertebrates) (52), which plays a crucial and central part in the anti-DENV immune response and many other viral infections. Upon virus recognition by PRRs, the IFN system is rapidly triggered within hours from infection, initiating IFN production and a cascade of signaling pathways (53), and as shown here, the transcription of an array of IRGs. In this context, this study comprehensively characterizes the dengue interferome signature in patients, encompassing different datasets, disease phases, and severities. The IFNs are among the oldest known cytokines (51) and the results presented here indicate that the interferome can still be explored in more detail in DENV-infected patients to provide new contextual information that may benefit these patients and result in improved treatment strategies. Our findings align with previous studies highlighting the significant role of IFN responses in the immune response to DENV infection (54–56). Indeed, we consistently observed a functional enrichment of several IFN-related functions during the acute disease phase. Thus, our integrative systems biology analysis based on seven independent datasets confirms the consistency of prior individual studies, such as those reported by Sun et al. (5), characterized by cytokine-mediated signaling (e.g., type I IFN) and chemotaxis, which is followed by a transcriptional wave of genes associated with the cell cycle.

Our current study emphasizes the predominance of IFN-regulated genes among DEGs, characterizing the interferome signature as an evident hallmark of the acute response to DENV. This finding agrees with the observation of high levels of type I IFN during acute dengue infection with concomitant CD4+ and CD8+ T cell activation at symptom onset (57). It is already well established that the first powerful wave of type I IFNs may be an early attempt of the host to protect itself against the initiation and development of more advanced disease by employing an early acute phase. Meanwhile, the enrichment of cycle-associated BPs at the late acute phase might represent the simultaneous occurrence of leukocyte proliferation to fight against the infection, together with the manipulation of the cell cycle by the DENV (58, 59) through distinct mechanisms that remain to be explored further in future studies. Moreover, our work demonstrates that IRGs are still differentially expressed in the late acute phase. As a result, as IFNs may still be detected, they may also play a regulatory role in this phase of the disease. Genes upregulated at the late acute phase enriched BPs mostly related to mitosis and cell cycle, which was also reported by Sun et al. (5), suggesting this may represent a recovery of the immune cells after viremia has decreased. Thus, indicating a long-lasting cascade effect of the interferome in the anti-DENV immune response.

Clinical investigations of dengue patients have assessed varying timeframes of IFN kinetics and levels during infection, e.g., higher IFN-α levels were identified in the period comprising the early acute phase (0 to 3 days after fever onset) (54, 60), but also throughout the acute phase (57). IFN-γ was also reported to be elevated in the early acute phase (57), although peaking around defervescence (61). Likewise, in the early acute phase, we observed the enrichment of BPs primarily related to antiviral defense mechanisms. These BPs included regulating typical viral processes, replication, life cycle, and signaling pathways of RLRs, as well as inflammatory cytokines. These results are consistent with the well-established functions of IFNs in innate immunity. Therefore, our integrative findings from different patient datasets confirm the consistency of the molecular dynamics of the early acute phase of dengue infection, when the viremia, innate immune responses, and IFN responses peak (52).

The observed cell-cycle-related effects of the distinct anti-DENV interferome signature should be investigated further. They may be explained by the fact that IFNs have been reported to negatively regulate the proliferation and differentiation of cell types, such as innate lymphoid type 2 cells and dendritic cells (53), as means of controlling the infection by hampering the viral replication and enhancing the elimination of infected cells (52). On the other hand, both IFN-α and IFN-γ have also been reported to enhance immune proliferation (62, 63). These apparently contradicting effects illustrate how the IFN system is highly pleiotropic, presenting extensive functions and effects (62, 64).

The stratification and classification analyses (PCA and random forest) results demonstrate effective discrimination between disease severities based on the interferome signature only in the late acute phase, indicating that the interferome has a more prominent role at this stage. The late acute phase is critical, wherein the disease course is defined as severe or non-severe dengue (4). So far, identifying early-stage biomarkers to classify which patients will progress to severe dengue is still an unsolved challenge. The random forest algorithm identified the top ten ranking genes (*IFI27*, *SULF2*, *ISG15*, *NME1*, *CYBRD1*, *GINS2*, *GAS6*, *UBE2T*, *RNASE1*, *MKI67*) for differentiation of DHF and DF in the late acute phase. Among them, *IFI27*, *ISG15*, and *CYBRD1* were also meta-significant genes. We confirmed in an independent dataset that these putative biomarkers are differentially expressed during acute infection for both DF and DHF and return to baseline expression upon disease resolution. Further, *IFI27* and *ISG15* were significantly upregulated in the DHF late acute phase and have previously been associated with dengue severity by Zanini et al. (65).

Mechanistically, *IFI27* (interferon alpha inducible protein 27) is involved in type-I interferon-induced apoptosis (49) and was recently identified as a key ISG in DENV infection, using similar approaches (66). In addition, *IFI27* was predominantly upregulated across disease phases in DHF and DSS, being a putative late-stage biological indicator for severe dengue. In turn, *ISG15* encodes a ubiquitin-like protein with an antiviral activity that can induce NK cell proliferation, act as a chemotactic factor for neutrophils, and induce IFN-γ (49). *ISG15* was upregulated in DHF but downregulated in DSS, representing a potential molecule differentiating patients between the two severities in the late acute phase. On the other hand, *CYBRD1* encodes a plasma membrane reductase that reduces extracellular Fe^3+^ into Fe^2+^, expressed in monocytes and neutrophils (49, 67). It is a meta-significant gene, downregulated in DHF but upregulated in DSS. *CYBRD1* can potentially be a classifier between DHF and DSS in the late acute phase, and to the best of our knowledge, it has not been previously associated with dengue. *GAS6* encodes a ligand for tyrosine-protein kinase receptors, which has been shown to bind to TAM receptors, inhibiting inflammatory innate immune response. DENV exploits the apoptotic clearance function of TIM and TAM receptors, mediated by Gas6, to gain entry into cells (68). This fact highlights the interplay between *GAS6* and DENV infection, emphasizing the involvement of *GAS6* in modulating immune responses and facilitating viral entry. As this gene was upregulated in the DHF group, this enhanced viral entry may be related to the increased disease severity.

Our work has some limitations. For instance, the molecular signatures identified in this study must be investigated in longitudinal studies and verified further at the protein level. As with all transcriptomic data, it does not necessarily reflect in expressed proteins or functional effects, and possible biomarkers are only putative, with further clinical studies necessary to evaluate their applicability. Another limitation is that some samples included children and infants, whose immune response differs from that of adults (69). As expected, most severe dengue (DHF and DSS) samples were secondary infection cases (70). However, no information allowed us to consistently identify the samples regarding either reinfection or DENV serotype, which can affect the response to infection (71). Other populational factors affecting the response to DENV that could not be considered in this study are ethnicity, geographic location, nutritional status, and comorbidities (72, 73), as that information were unavailable and/or not comparable. Despite these limitations and the cohort heterogeneity, we found consistent molecular signatures across the studies, indicating our results are robust and characteristic of the overall DENV infection.

## 5 Conclusions

Our study underscores the significant involvement of IFN-regulated genes in the acute dengue response, as reflected by the distinct interferome signature during different phases of DENV infection. Summarily, our approach focused on the expression patterns of IRGs across disease phases and severities in dengue patients. Of note, most DEGs induced by dengue infection were also regulated by IFN, which corroborates the broad modulation of the immune response by the IFN system. Moreover, this study provides valuable insights into the qualitative and quantitative aspects of the dengue interferome, highlighting the dynamic interplay between IFN signaling and gene expression modulation in the context of dengue infection. Hence, our study indicates consistent molecular signatures of disease severity in the late acute stage that can help the development of better classificatory methods and treatment to reduce morbidity and mortality of dengue patients.

## Data availability statement

Publicly available datasets were analyzed in this study. This data can be found here: GEO Datasets (https://www.ncbi.nlm.nih.gov/gds), accession numbers GSE25001, GSE28405, GSE28988, GSE28991, GSE43777, GSE40628, GSE51808. Codes utilized in this article are available at the following link: https://github.com/JNUsuda/dengue_interferome.

## Ethics statement

Ethical approval was not required for the study involving human samples in accordance with the local legislation and institutional requirements. Written informed consent for participation in this study was provided by the participants’ legal guardians/next of kin. Ethical approval was not required for the study involving animals in accordance with the local legislation and institutional requirements because publicly available datasets were used in this study.

## Author contributions

JU, DP, and OC-M conceived this study. JU performed the data analysis. DF, AM, IF, VC, AA, AT-C, MH, PF, RC, GM, GC, LS, DP, and OC-M provided scientific insights. JU, RC, GM, DP, and OC-M wrote and revised the final version of the manuscript. DP and OC-M supervised the work. All authors contributed to the article and approved the submitted version.
