# Supplementary material for: Interferome signature dynamics during the anti-dengue immune response: a systems biology characterization
Source: Front Immunol. 2023 Aug 10;14:1243516. doi: 10.3389/fimmu.2023.1243516 (PMC10449254; doi:10.3389/fimmu.2023.1243516)
Supplement: Supplementary file 1 [file DataSheet_1.docx]

Supplementary Material

Interferome signature dynamics during the anti-dengue immune response: a systems biology characterization

Júlia Nakanishi Usuda*, Desirée Rodrigues Plaça*, Dennyson Leandro M. Fonseca, Alexandre H.C. Marques, Igor Salerno Filgueiras, Victor Gabriel Bastos Chaves, Anny Silva Adri, Amanda Torrentes-Carvalho, Mario Hiroyuki Hirata, Paula Paccielli Freire, Rusan Catar, Guido Moll, Gustavo Cabral-Miranda, Lena F. Schimke, Otavio Cabral-Marques*

*** Correspondence:**
Júlia Nakanishi Usuda: julia.usuda@usp.br
Desirée Rodrigues Plaça: desiree.placa@usp.br
Otavio Cabral-Marques: otavio.cmarques@usp.br

# Supplementary Figures

**Figure S1.** DENV infection acute phase interferome across disease severities.

**Figure S2.** Top interferon-regulated genes for severity classification ranked by random forest (Dataset A).

**Figure S3.** Early acute phase random forest error rates and ROC curves.

**Figure S4.** Expression of putative biomarkers for predicting severe dengue clinical outcome classification across disease phases.

## Supplementary Figure 1


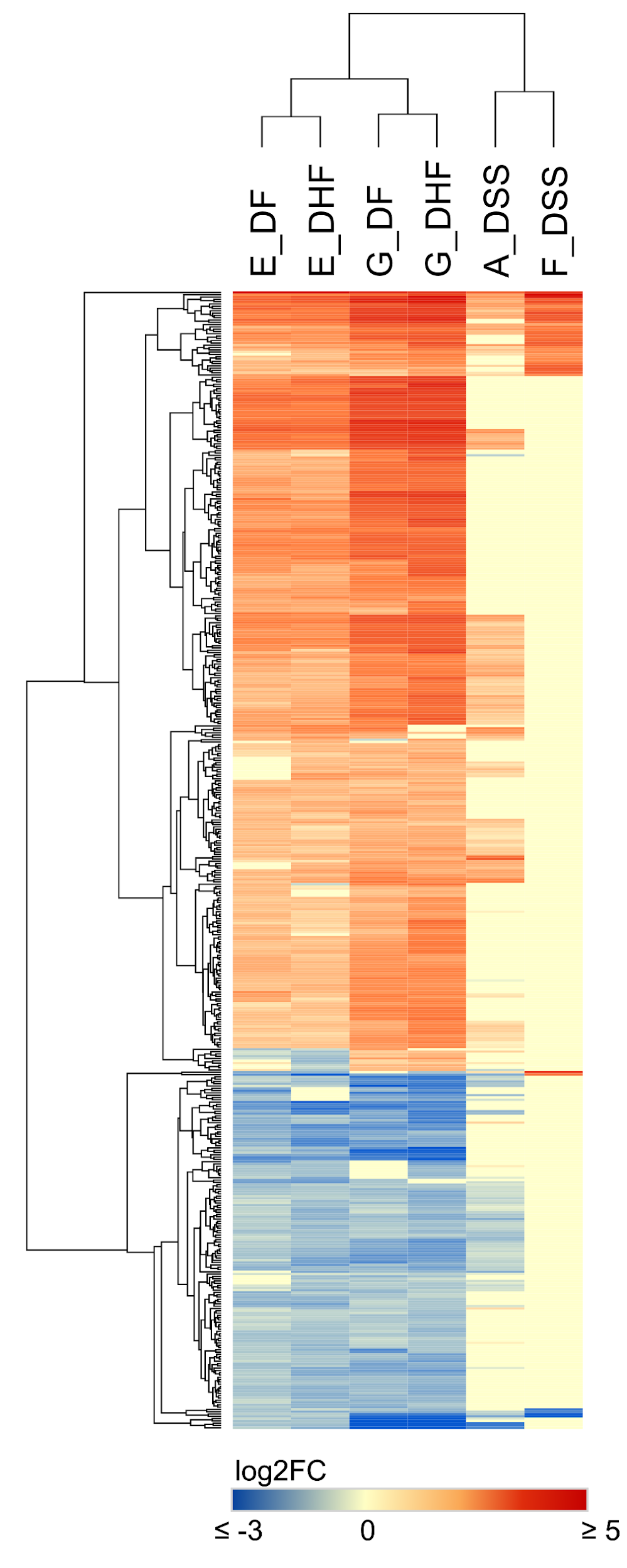


**Figure S1. DENV infection acute phase interferome across disease severities.** Heatmap of log2 FC of common IRGs across datasets with hierarchical clustering by Euclidean distance (**Table S9**). The red scale indicates upregulated genes, the blue scale indicates down-regulated genes, yellow indicates genes missing or with FC close to zero. Heatmap columns legend: First letter indicates datasets (A, GSE25001; E, GSE43777; F, GSE40628; G, GSE51808), while the second term indicates disease severity comparisons (DF: acute dengue fever vs. convalescent; DHF: acute dengue hemorrhagic fever vs. convalescent; DSS, acute dengue shock syndrome vs. convalescent). *DENV, dengue virus; FC, fold change; IRG, interferon-regulated gene.*

## Supplementary Figure 2


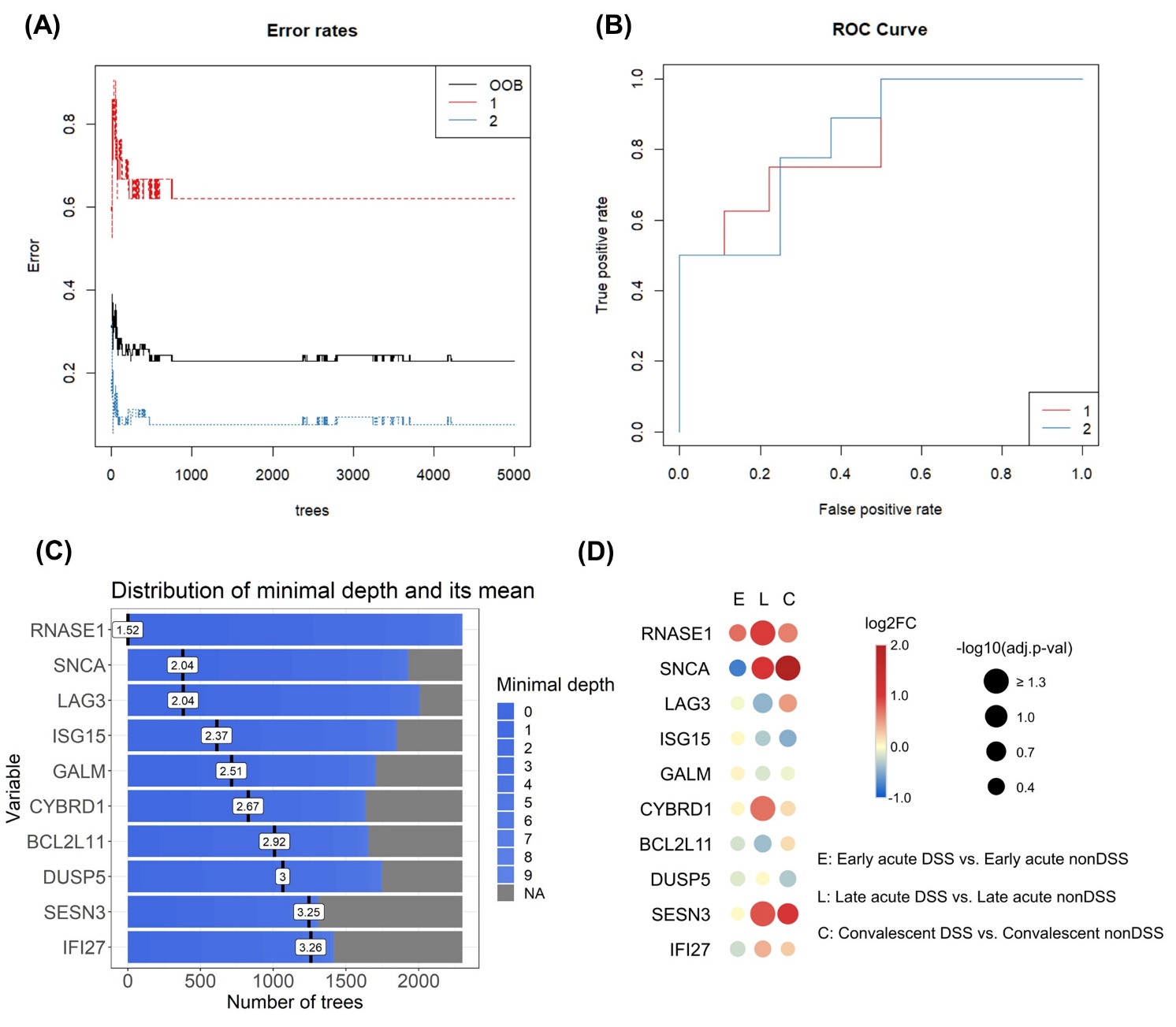


**Figure S2. Top interferon-regulated genes for severity classification ranked by random forest (Dataset A). (A)**, Error rates of random forest models by number of trees. **(B)**, Receiver operating characteristic (ROC) curve of the generated classifying models. The red line corresponds to group 1 (late acute nonDSS), blue line corresponds to group 2 (late acute DSS). **(C)**, Bar plot of the top ten severity classifying genes ranked by the random forest model, number of trees, and distribution of the minimal depth. Blue bars represent the minimum and maximum minimal depth, and black vertical lines represent the mean minimal depth for each classifying gene. Data input of log2-transformed expression values of IRGs common across datasets A to E in the late acute phase is available in **Table S13**. **(D)**, Bubble heatmap of the log2FC of IRGs resulting from the DSS vs. nonDSS comparison for each disease phase (early acute, late acute, convalescent). The red scale indicates positive FC (up-regulated genes), blue scale indicates negative FC (down-regulated genes). Bubble size represents -log10-transformed adjusted p-value. Data is available in **Table S14**. *IRG, interferon-regulated gene; DF, dengue fever; DSS, dengue shock syndrome; FC, fold chang*e; *OOB: out-of-bag.*

## Supplementary Figure 3


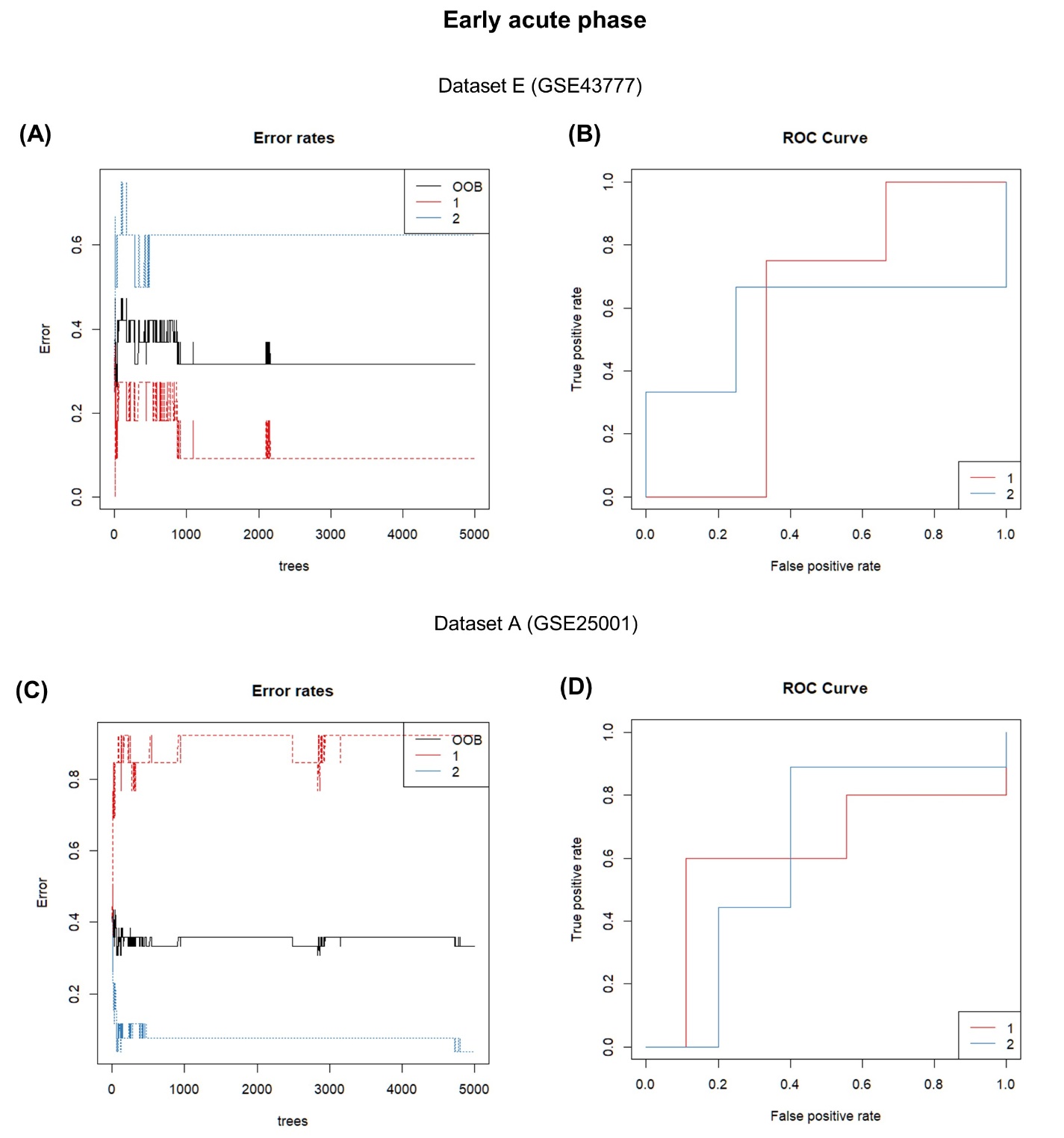


**Figure S3. Early acute phase random forest error rates and ROC curves. (A)**, **(C)**: Error rates of random forest models by number of trees. **(B)**, **(D)**: Receiver operating characteristic (ROC) curve of the generated classifying models. Data input of log2-transformed expression values of IRGs is available in **Table S13**. Dataset E: red line corresponds to group 1 (early acute DF), blue line corresponds to group 2 (early acute DHF). Dataset A: red line corresponds to group 1 (early acute DSS), blue line corresponds to group 2 (early acute nonDSS). *IRG, interferon-regulated gene; DHF, dengue hemorrhagic fever; DF, dengue fever; DSS, dengue shock syndrome; FC, fold chang*e; *OOB: out-of-bag.*

## Supplementary Figure 4

*
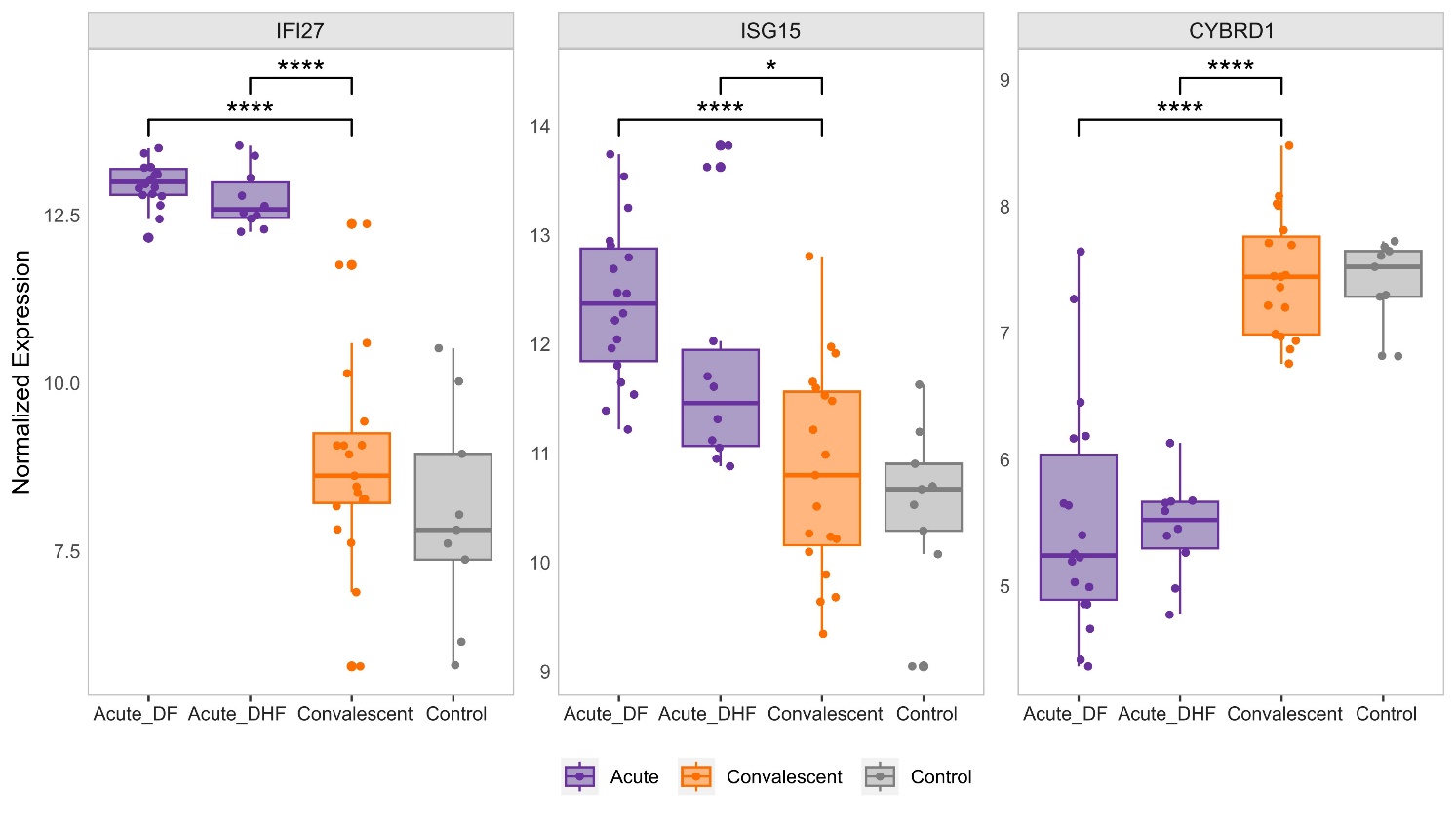
*

**Figure S4. Expression of putative biomarkers for predicting severe dengue clinical outcome classification across disease phases.** Box plots represent the median and interquartile range of Robust Multi-array Average (RMA)-normalized and mean-summarized expression of genes *IFI27*, *ISG15,* and *CYBRD1* in the DF or DHF acute phase (purple), convalescent (orange), and healthy control (gray) samples from dataset G (GSE51808) (**Table S16**). Wilcoxon’s test between acute DF or acute DHF and convalescent groups indicated statistical difference (stars indicate p-value: * p ≤ 0.05; **** p ≤ 0.0001). *DHF, dengue hemorrhagic fever; DF, dengue fever.*
